# The Human Body: Its Plan of Organisation

**Published:** 1916-04-22

**Authors:** 


### THE HUMAN BODY.

### Its Plan of Organisation.

Not infrequently, and more especially perhaps in popular lectures, the human body is compared to a machine. It has a certain structure and arrangement of parts or organs, and each of these has some subordinate value or function in relation to the whole. Fuel in the shape of food is introduced into the machine and is burned or consumed under the influence of oxygen taken in through the lungs. The results are seen in the shape of energy or work, such as bodily heat and movement. As a consequence of the combustion processes on which this energy depends, waste products, comparable to the smoke and cinders from a fire, are formed, and these are expelled from the body through the kidneys and other organs. And the co-ordination of the various parts is secured by the governing agency of the nervous system. Such, in brief outline, is a picture which is not without its value for the student who proposes to take an amateur interest in human physiology. Whatever its merits, however, the analogy has one capital error. It suggests that the various parts and organs of the body are made to work by the agency of a power external to themselves. There is a mechanism and a driving force, and by the latter the several pieces of the machine are made to perform their functions. The food undergoes combustion and the wheels turn round. Now all this is, of course, inadequate or even misleading. The truer statement is that every active part of the body works by its own initiative, not that it is merely made active by some outside force. It accomplishes its function not as a mechanical structure under the play or pressure of some power external to itself, but by the active operation of its own inherent powers. There is effort from within, rather than impulsion from without, and this applies to each of the individual tissues and organs of which the body is composed. Hence the comparison of the body to a machine fails in an essential feature. A more adequate comparison is that of a colony of living beings, each member of which has its own individual necessities and each of which, in addition, has some duty to discharge in relation to the whole. So long as these necessities are met, and so long as these duties are performed, there is ease and peace in the colony; the body is in a state of health. When these conditions are not satisfied, there is more or less disturbance, disability, or disease.

It is, of course, literally true to say that the human body is a colony of living organisms. Every tissue or organ displays to the microscope multitudes of cells or corpuscles, each one of which is within measure an independent vital unit. The wants of these vital units are, in principle, the same as those of the free and detached units which are the most humble forms of life, and essentially both the one group and the other possess identical properties. Thus, so long as they are alive they successfully resist the disintegrating influences which

### The Human Body *(continued)*.

surround them, which means that even when they appear quiescent they are really displaying energy—the energy of resistance. Change their surroundings and environment and they will show an active response to the change—it may be an alteration of position or of form or some other conspicuous display of energy. That is, the living cell or corpuscle has the power, as the physiologist phrases it, to respond to a stimulus.

### Food and Waste Products.

And again, the living cell may, under certain circumstances, detach a portion of its substance to become a new living being like unto itself (reproduction). These various activities determine the conditions which are necessary in order that living cells may continue to manifest energy, that is may continue to live. As every display of energy means chemical change in the substance of the corpuscle, this is continually being wasted or broken down. Consequently there is need for the supply of new material in the shape of food, which the corpuscle takes into itself and out of which it builds up new substance to repair the waste and to provide for growth. A second condition is a supply of air or oxygen, without which the chemical changes essential to the manifestation of energy are impossible, while, in the third place, as each manifestation of energy means breaking down of the substance of the corpuscle, there is a continual production of waste materials, and these being useless, as well as hurtful to the activity of the corpuscle, must be expelled. A supply of food, a supply of oxygen, and the removal of waste materials—such are the essential necessities of each living cell, and this applies whether the cell is a completely independent microscopic creature, or whether it is mechanically tied to others, as in the colony which is known as the human body. In the one instance the entire organism consists of a single cell, in the other of many cells. But in each alike the wants and necessities of every individual cell remain as above defined.

Now, for a uni-cellular organism living, as is usually the case, in water, these essential conditions are readily satisfied. It finds floating in the water the materials it needs as food. Similarly, dissolved in the same medium is sufficient oxygen for its needs. And again, into the water it expels its waste. Further, fully to meet these elementary wants, it may move from one place to another. But the position of each individual unit in the case of a multi-cellular organism is very different from that just described. Here the individual cell is not free to move independently of its neighbours. On the contrary, it is bound to others by a chain which cannot be broken. In crude language, it cannot go in search of food or oxygen, and the waste matters which it expels will be expelled to the prejudice of its fellows. Yet the prime necessities of cellular life and activity remain, and somehow or other they must be satisfied. The question is, How are these to be met in an organism which consists, not of a single cell, but of many cells? To answer this question in detail would be to write a volume on physiology. Yet the principle of the plan adopted is comparatively simple, and the statement of this principle is what is here to be attempted.

### The Circulation.

In the first place, there is carried throughout the colony a series of tubes, the blood-vessels, which contain a nutrient fluid, the blood. The finest of these tubes, the capillaries, have such delicate walls that some of the fluids of the blood pass through them and surround the neighbouring corpuscles; and the distribution of the capillaries is so abundant that no corpuscle is far away from the blood supply. Consequently every corpuscle is bathed by nutrient fluid, and from this fluid it takes the food and oxygen it needs and into this fluid it throws its waste matters. Thus it is literally true to say that the vital units of the colony live on the banks of the nutrient stream contained within the capillary blood-vessels, and it is from this stream that their essential necessities are met. But obviously, as the millions of living units of which the colony is composed are taking food and oxygen from the fluid which surrounds them, the value of this fluid will soon become exhausted; and similarly, if these units are expelling waste materials into this fluid, it will promptly be rendered unsuitable for its purpose. Consequently there is the necessity to add fresh supplies of food and oxygen to the blood, and to remove from it accumulated waste materials. The system of blood-vessels is therefore associated with other mechanisms devoted to these several ends. Running through the centre of the colony, as it were, is a tube, the alimentary canal, into which supplies of food are regularly introduced. Here the food materials are digested or prepared for entrance into the blood, and through the walls of some parts of the alimentary tube—the stomach, for example—the digested food does, as a fact, enter this fluid. In this fashion the nutrient materials taken from the blood by the colony units are replaced by new supplies. Similarly, through another tube, the wind-pipe, air, containing a certain proportion of oxygen, reaches two elastic bags, the lungs, and from these gains admission into the blood contained in delicate vessels lining the walls of these bags. Thus is secured the new supply of oxygen which the blood needs to replace the oxygen used up by the living cells of which the body is everywhere composed.

There now arises a fresh necessity. Clearly the fact that there is food in abundance in the blood in the neighbourhood of the stomach will be of no value to a corpuscle situated in some remote part of the body; and to the same corpuscle equally futile will be the presence of rich supplies of oxygen in the blood in the neighbourhood of the lungs. To be of value to any such living unit the blood which contains these added materials must be brought within its reach. There must, in other words, be a movement or flow of the blood from the districts in which new materials enter it to the parts where these materials are needed by the living cells, that is to say there must be blood-flow throughout the whole length and breadth of the colony. Not only must the blood be universally present in all parts of the colony, not only must new food materials and oxygen be added to it, but, in addition, there must be a circulation of the blood so that its values may be placed within the grasp of each one of the living cells. Hence a certain group of corpuscles is told off to form a muscular pump, the heart; others construct distributing tubes, the arteries, which conduct the blood from the heart to the capillaries; and a third set form the veins which carry the blood back from the capillaries to the heart. Then by the strokes or contractions of the heart the blood is driven along the arteries, through the capillaries and back by the veins, the direction of the flow being secured by certain valves inserted in the heart and in some of the vessels. As it passes through the capillaries it yields the food and oxygen which the corpuscular units need, while as it travels through the walls of the alimentary canal and of the lungs new supplies of these materials are added. In the course of the circulation, too, the blood is brought to other physiological mechanisms, such as the kidneys, whose duty is to drain off from it the waste materials which it has received from the tissues.

### The Nervous System.

But if all this scheme of organisation is to work smoothly and successfully, there must clearly be some controlling agency, and some method of communication between the several parts of which it is composed. What is seen is a vast colony of living units, the members of which have their elementary needs supplied from the blood. But in addition to its duty of keeping itself alive, each living unit has some duty to the colony as a whole. One group, as we have already seen, has to drive the blood along the vessels, another to form materials necessary for the digestion of the food, and a third to extract waste matters from the blood. Similarly, to others are allotted the duty of building and maintaining the fabric of the bones; the responsibility of moving the body falls to the muscles; while on the surface of the colony are certain corpuscles whose function it is to become active on the approach of danger. There is, in short, a division of labour in the colony, and each group of corpuscles is under a particular obligation to its fellows. To discharge that obligation there must be opportunity for inter-communication. Taking a simple example, the cells on the surface which have to note the approach of danger must be able to signal to the muscle cells whose duty it is to move the body out of harm's way. To meet this need there run throughout the body a series of filaments or nerves, along which, as along telegraph-wires, messages may travel. These filaments all converge to a great collection of corpuscles known as nerve-centres, the great majority of which are collected in the brain and spinal cord, and here in-going and outgoing messages are continually in operation. The nerve-centres form as it were a great central bureau for the reception and distribution of the demands and announcements of the various parts of the colony, and in this way all the activities of the corpuscular groups are kept in harmonious adjustment.

Such in principle is the physiological plan of the human body. Out of this plan—and here is the main purpose of the scheme—there arises the existence of a group of corpuscles which are, as it were, so free from other demands that they develop the possibility of responding not merely to chemical and mechanical stumuli, but also to intellectual, ethical, and æsthetic influences. Indeed, in this development is found the physiological justification of the existence of the human body. Isolated, independent, detached vital units must necessarily occupy the whole of their energies in the satisfaction of their primitive wants. But with the union of multitudes of cells into a great colony, and with the application in this colony of the principle of the division of labour, there is gradually evolved a series of corpuscles whose nutritive necessities are met by a mere minimum of effort. Hence these are able to develop capacities above and beyond those of their fellows; they are touched by and respond to influences which stir no activity in units occupied solely with their own material needs or with such duties as the building of bones or the propulsion of the blood. Of course, without the work of these latter groups the nerve centres of higher capacity could not exist, and it is in order that they may exist that all the other members of the colony carry on their subordinate and relatively lowly labours. That in the colony there may be members free to develop the ability to respond to influences other than those of the material world, there must needs be hewers of wood and drawers of water; and all of these merit due honour. They take their part in a scheme of things which finds its explanation not in themselves, but in developments which they help to render possible. The explanation comes in the end, when are produced members of the colony whose special function it is to be active under the claim of duty and the demand of truth and the force and stimulus of moral law. Without union of vital units to form a colony, the development of such powers in corpuscles is not possible, and it is in their development is found the interpretation and secret of the whole scheme of bodily organisation. Yet it remains true that in corpuscles boasting these capacities, even as in others, the primary necessities of vital units still remain, and it is to satisfy these necessities that the organs of digestion, of respiration, of circulation, and of excretion exist and work. "Through the ages an increasing purpose runs," and it is literally true to say that the capacities of corpuscles, even as the thoughts of men, "are widened with the process of the suns." An essential condition of this achievement is that vital units shall be gathered into colonies on an organised plan, and when this is appreciated the complexities of tissues and organs find their justification and sanction.

